# High-grade large cell neuroendocrine carcinoma of the esophagus: a case report and review of the literature

**DOI:** 10.1186/s13256-023-03879-0

**Published:** 2023-04-07

**Authors:** Hussein Awada, Adel Hajj Ali, Ahmed Bakhshwin, Hamed Daw

**Affiliations:** 1 Department of Translational Hematology and Oncology Research, Taussig Cancer Institute, Cleveland Clinic, Cleveland, OH 44195 USA; 2 Heart, Vascular & Thoracic Institute, Cleveland Clinic, Cleveland, OH 44195 USA; 3 Pathology Department, Robert J. Tomsich Pathology & Laboratory Medicine Institute, Cleveland Clinic, Cleveland, OH 44195 USA; 4 Hematology and Oncology, Cleveland Clinic Fairview Hospital, Cleveland, OH 44111 USA

**Keywords:** Neuroendocrine tumor, Large cell carcinoma, Esophageal cancer

## Abstract

**Background:**

Neuroendocrine carcinomas are extremely rare in the esophagus as they represent less than 0.04% of all neuroendocrine tumors. To date, only 14 cases of poorly differentiated, high-grade esophageal NEC have been described in the literature. The majority of these patients presented with typical dysphagia symptoms. Due to its rarity, no standardized guidelines have been proposed to treat esophageal neuroendocrine carcinoma, although general recommendations suggest surgery with adjuvant chemoradiotherapy as the treatment of choice.

**Case presentation:**

A 67-year-old previously healthy White male presented with a year-long intermittent nonspecific retrosternal discomfort, with the absence of any other symptoms. Esophagogastroduodenoscopy revealed an ulcerative mass in his lower esophagus, with concern of malignancy. Endoscopic ultrasound-guided biopsy revealed poorly differentiated neuroendocrine carcinoma of the esophagus with metastasis to a diaphragmatic lymph node. He was treated with neoadjuvant chemoradiation followed by surgery, and he has been in remission for over 5 years.

**Conclusion:**

Here, we review the literature and report a unique case of a patient with a vague presentation of esophageal neuroendocrine carcinoma as he enters his sixth year of survival following neoadjuvant chemoradiotherapy.

## Introduction

Esophageal cancer is currently the eighth most common malignancy and the sixth cause of cancer-related deaths worldwide [1]. Tumors of the esophagus are predominantly of squamous cell or adenocarcinoma histology; however, other types such as stromal tumors, leiomyomas, leiomyosarcomas, as well as neuroendocrine neoplasms exist, although their incidence is rare. Esophageal neuroendocrine carcinomas (NECs) represent 0.04% of all NECs at all anatomical sites and 0.4–2.0% of all esophageal malignancies [2, 3]. They have an incidence rate of 0.44 per million-year [4]. NECs can be of either small cell or large cell morphology, with the former representing around 90% of all NECs [3]. Large cell esophageal NECs are typically associated with rapid growth and poor prognosis [5]. Standardized guidelines for the treatment of these neoplasms remain lacking as the rarity of these tumors hinders the development of large cohort studies that provide evidence-based recommendations. Here, we describe the case and management of a previously healthy 67-year-old male in his fifth year of remission after treatment for poorly differentiated large cell neuroendocrine carcinoma of the esophagus.

## Case presentation

**Fig. 1:**
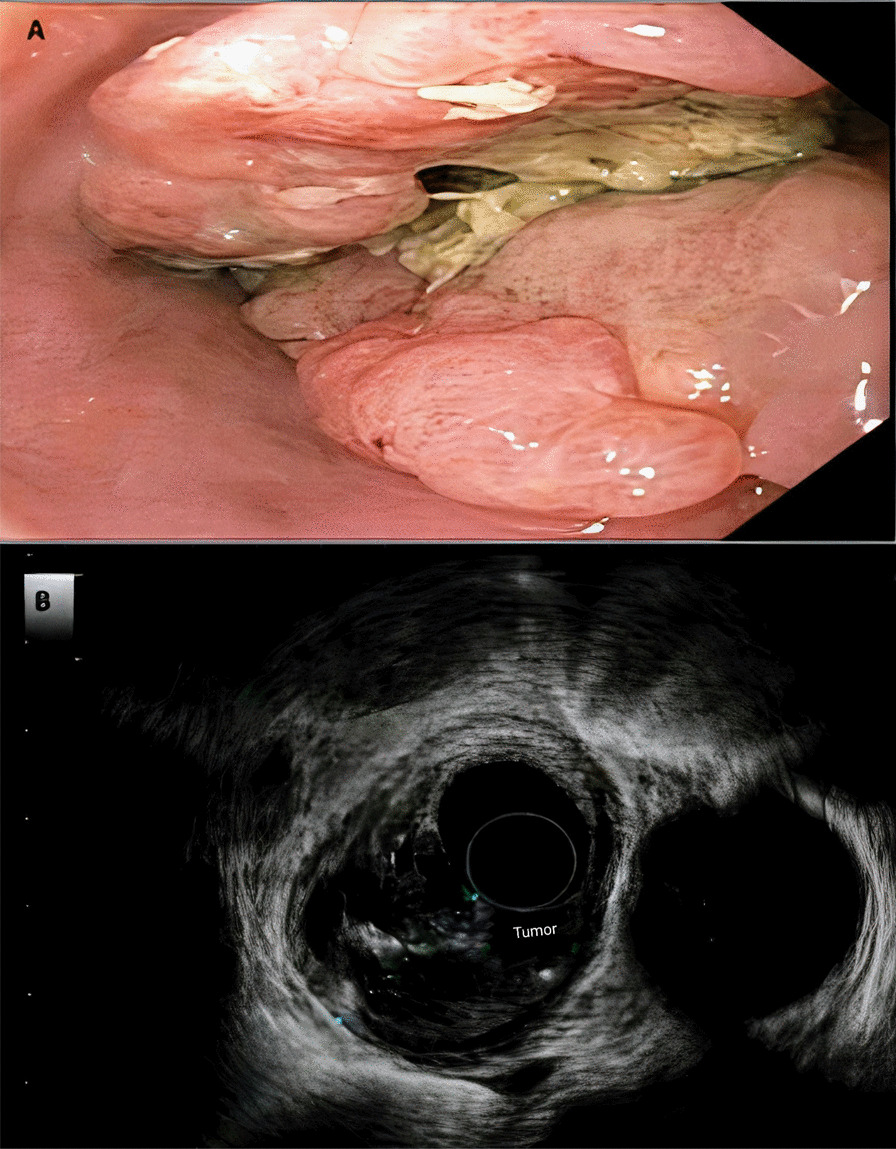
**A** Esophagogastroduodenoscopy and **B** endoscopic ultrasound showing the tumor

A 67-year-old White male with no past medical history presented to the gastroenterology clinic in February 2017 with a complaint of persistent intermittent mid-chest discomfort over the past year. Prior to presentation, the patient underwent a negative coronary workup for intermittent mid-chest discomfort of unclear etiology. The patient described a retrosternal chest pressure that only later became associated with food intake. He denied heartburn, regurgitation, dysphagia, nausea, vomiting, abdominal pain, bleeding, dyspepsia, fevers, dark stools, night sweats, or weight loss. The patient did not use any medications, was never a smoker, did not drink alcohol, and denied a significant familial medical history. On physical exam, the patient appeared in no acute distress and had a normal exam of his oral cavity, along with a soft, non-tender, non-distended abdomen and no apparent organomegaly or lymphadenopathy. His initial complete blood count, basic metabolic panel, and liver function tests were within normal ranges. His negative workup and persistent symptoms, which were refractory to conservative measures, warranted further evaluation. He was referred for endoscopy, which revealed an ulcerative mass and a stricture in the distal third of his esophagus—concerns for malignancy (Fig. 1A).

**Fig. 2:**
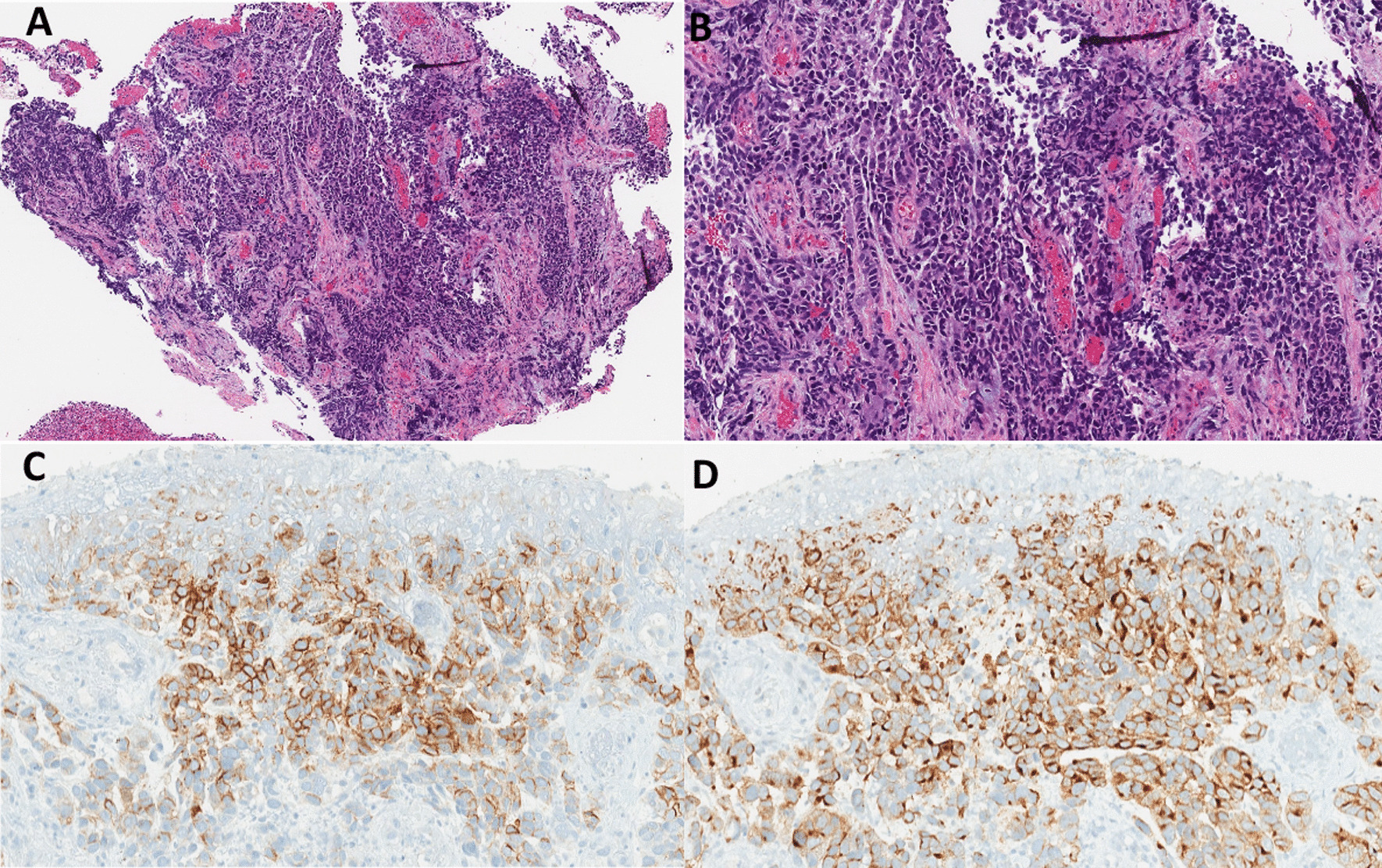
**A** On low magnification, the biopsy showed islands of tumor separated by fibrovascular septae [hematoxylin and eosin (H&E) ×4]. **B** The neoplastic cells have large, hyperchromatic nuclei, irregular nuclear membranes, inconspicuous nucleoli, and scant amount of amphophilic cytoplasm (H&E ×20). The presence of significant nuclear molding on the right side of the image was also noted. The tumor cells demonstrate strong and diffuse expression of CD56 (**C**) and synaptophysin (**D**)

Endoscopic ultrasound (EUS) examination further characterized the mass as 5 cm in length, 16–18 mm in thickness, and as a partially circumferential mass contained within the submucosal area, with evidence suggesting muscularis propria invasion (Fig. 1B). It was staged T3N0M0 as per endosonographic criteria. The mass appeared to be primarily below the diaphragmatic hiatus and its center within 0.5 cm of the gastroesophageal (GE) junction. Positron emission tomography (PET) scans demonstrated fluorodeoxyglucose (FDG) avidity involving the GE junction and extending to the lesser junction, but no signs of FDG avidity elsewhere. Biopsy obtained from EUS suggested a poorly differentiated carcinoma of suggested large cell neuroendocrine histology (Fig. 2). As such, immunohistochemical staining followed, and it showed strong immunoreactivity for both synaptophysin and CD56, but showed negative chromogranin immunoreactivity. Serum chromogranin A was mildly elevated at 16 ng/ml (normal < 15 ng/ml), while serum serotonin at 154 ng/ml and 24-h urine 5-hydroxyindoleacetic acid (5-HIAA) at 3.1 mg were normal (normal ranges < 230 ng/ml and < 8 mg/24 h, respectively). These findings confirmed the diagnosis of a stage IIA poorly differentiated large cell neuroendocrine tumor.

The patient was started on neoadjuvant chemoradiation in June 2017, consisting of weekly carboplatin with target area under the curve 2 (AUC2), and paclitaxel 50 mg, along with radiotherapy, ahead of surgical resection. He tolerated four cycles of chemotherapy and seven sessions of radiotherapy, which helped in shrinking his tumor size. This was followed by a successful and noncomplicated robotic-assisted, laparoscopic esophagectomy in July 2017. Surgical biopsies and lymph node dissection revealed metastatic involvement in 1 of 3 diaphragmatic lymph nodes and no tumor invasion beyond the muscularis propria, further fine-tuning staging as T2N1M0. The patient has been under active surveillance, with yearly EGD and computed tomography (CT) scans every 6 months.

## Discussion

Neuroendocrine neoplasms of the esophagus are generally classified by their grade, in which low and intermediate-grade neoplasms are labeled as G1 and G2 neuroendocrine tumors (NETs), respectively. In contrast, high-grade neoplasms are categorized as NECs. Their incidence is even more scarce when characterized by a high-grade, large cell histology, with only 11 out of a total of 14 patients with such confirmed similar histology reported in the literature thus far (Table 1) [6–17]. The majority of these patients (including our patient) were males (80%), and their median age at diagnosis was 67 years (range 42–85 years).

**Table 1:** Review of all cases of poorly differentiated, large cell esophageal neuroendocrine carcinoma

Patient	Age	Gender	Smoking	Location	Stage	Presentation	Treatment	Survival (months)
1 [6]	83	M	N/A	Upper	IVA	Dysphagia	Palliative	< 2
2 [7]	58	M	N/A	Lower	IIB	Dysphagia	Adjuvant cisplatin + etoposide	> 36
3 [8]	73	M	Current	Lower	IVB	Dysphagia	Adjuvant docetaxel + fluorouracil	20
4 [9]	51	M	Never	Lower	IA	Dysphagia	Adjuvant chemoradiotherapy	N/A
5 [10]	55	M	N/A	Lower	IVB	Abdominal pain	Palliative	1
6 [11]	81	M	N/A	Lower		Weight loss	Surgery	> 18
7 [12]	66	F	Never	Mid	IVB	Dysphagia	N/A	N/A
8 [13]	81	M	N/A	Mid		Dysphagia	Adjuvant chemoradiation	8
9 [14]	75	F	N/A	Lower	IIA	N/A	Adjuvant irinotecan + cisplatin + radiotherapy	13
10 [14]	60	M	N/A	Mid-lower	IIIB	N/A	Adjuvant irinotecan + cisplatin	13
11 [14]	69	M	N/A	Lower	IIIB	N/A	Neoadjuvant irinotecan + cisplatin + radiotherapy	18
12 [15]	43	M	Never	Lower	IVB	Abdominal pain	Fluorouracil + cisplatin	11
13 [16]	85	F	Second-hand smoking	Mid	IVA	Dysphagia	Palliative	1
14 [17]	65	M	N/A	Lower	IVA	Heartburn	Adjuvant chemotherapy	9

*M* Male; *F* Female; *N/A* Not available; *I, II, III, IV* cancer stages

NEC commonly originates in the mid to lower esophagus, and this is likely related to the higher presence of Merkel cells in the mid-esophagus, as well as that of endocrine cells in the cardiac glands of the distal section of the esophagus. Neuroendocrine neoplastic cells may be functional (that is, secrete serotonin and so on), yet the majority are nonfunctional, especially if they are of poor histological differentiation[18]. Moreover, the aggressive nature of NEC (including small cell), especially in the case of high-grade disease, confers a high percentage of metastasis (57%) at the time of initial evaluation [19]. This is further emphasized by the described cases of large cell NEC, among which half of the patients presented with stage IV malignancy (Table 1).

Although risk factors specific to esophageal neuroendocrine neoplasms are not very well defined, smoking and alcohol consumption are accepted as the main accomplices, similar to esophageal squamous cell carcinoma [20]. In addition, esophageal NEC has been suggested to be associated with a prior history of achalasia, gastroesophageal reflux disease, and Barrett metaplasia, similar to esophageal adenocarcinoma. However, our patient was previously healthy, with no family history of malignancy, as well as no personal history of smoking or alcohol consumption.

A review of the reported cases shows that dysphagia is the most commonly reported symptom on presentation (63.64%), yet clinical symptom presentation can still vary between classical generalized systemic (anorexia, fatigue, weight loss…) and gastrointestinal (nausea, emesis, GI bleeding….), to respiratory, hematological, or endocrine symptoms, depending on the location, size, spread, and secretory activity of the tumor. In addition, the diagnosis may be incidentally made upon endoscopic examinations [16]. In contrast, our patient had neither any generalized, nor upper or lower gastrointestinal, signs or symptoms. His only complaint was a nonspecific chest discomfort, which further complicated the diagnosis. Furthermore, his lack of dysphagia can be best explained by the location of his tumor below the diaphragmatic hiatus.

In contrast to the well-differentiated NET, the diagnostic workup of poorly differentiated NEC relies more on positron emission tomography (PET) scans, rather than serum markers such as 5-HIAA, chromogranin A or somatostatin receptor imaging. This is because the high-grade status of the latter results in fewer neurosecretory granules, which increases the likelihood of false negatives, and hence, diminishes the reliability of these markers. This has also been demonstrated in our patient, whose serum chromogranin A, serum serotonin, and 24-h urine 5-HIAA were either barely above or within the normal ranges.

Owing to its rarity, no consensus about the optimal treatment regimen and duration of esophageal NEC exists. In the biggest cohort study, Lee *et al.* proposed a treatment algorithm based on tumor size and lymph node metastasis [20]. Our patient had a tumor size of 5 cm (> 1 cm) with regional lymph node metastasis and would fall under the surgical resection with adjuvant chemotherapy ± radiotherapy or palliative chemotherapy treatment plan. In our case, he underwent neoadjuvant chemoradiotherapy prior to surgery, unlike what was suggested by Lee *et al.*; nonetheless, the patient has been in complete remission for over 5 years thus far, as compared with the median survival time of 27 months [20]. In addition, Lee *et al.* reported size > 2 cm as being the only significant negative prognostic factor, with the median survival time for this group almost half the median survival time for all the patients in his study.

Further review of the high-grade, large cell esophageal NEC cases in the literature asserts a median survival of 12 months (range 1–36 months). Only one of these patients underwent neoadjuvant chemoradiotherapy, as reported in the cohort by Tustumi *et al.*, which included three patients with large cell NEC [14]. Compared with the other two patients of that same cohort, the patient who received neoadjuvant chemoradiotherapy survived longer (18 months versus 13 months for each of the other two), despite being either of a more advanced disease stage (IIIB versus IIA), or older (69 vs 60) but with the same disease stage. Still, our patient has survived for a remarkably longer period, which extends beyond the survival range of all the reported cases, despite year-long symptoms prior to diagnosis. This highlights the need for larger-scale research to determine the ideal management and timing of chemoradiotherapy of this disease.

## Conclusion

In our case report, we presented the case and management of a patient with poorly differentiated large cell esophageal NEC, while also reviewing the literature for similar reported cases. Despite the highly associated mortality rates, the challenging diagnosis, and the lack of standardized treatment guidelines, our patient had a great outcome with an ongoing remission of more than 5 years, following neoadjuvant chemoradiotherapy that preceded surgical resection of the tumor. Extensive research is still needed to determine and standardize the optimal treatment modalities for high-grade large cell esophageal NEC.

## Data Availability

Upon request by the editor.

## Abbreviations

NEC: Neuroendocrine carcinoma
EUS: Endoscopic ultrasound
GE: Gastroesophageal
FDG: Fluorodeoxyglucose
PET: Positron emission tomography
5-HIAA: 5-Hydroxyindolacetic acid
AUC: Area under the curve
EGD: Esophagogastroduodenoscopy
CT: Computed tomography
NET: Neuroendocrine tumor

## References

[1] Sung H, Ferlay J, Siegel RL, Laversanne M, Soerjomataram I, Jemal A (2021). Global cancer statistics 2020: GLOBOCAN estimates of incidence and mortality worldwide for 36 cancers in 185 countries. CA Cancer J Clin.

[2] Modlin IM, Sandor A (1997). An analysis of 8305 cases of carcinoid tumors. Cancer.

[3] Egashira A, Morita M, Kumagai R, Taguchi KI, Ueda M, Yamaguchi S (2017). Neuroendocrine carcinoma of the esophagus: clinicopathological and immunohistochemical features of 14 cases. PLoS ONE.

[4] Li Z, Hu J, Chen P, Zeng Z (2020). Incidence, treatment, and survival analysis in esophageal neuroendocrine carcinoma population. Transl Cancer Res.

[5] Ma Z, Cai H, Cui Y (2017). Progress in the treatment of esophageal neuroendocrine carcinoma. Tumour Biol.

[6] Kubo T, Adachi Y, Ishii Y, Endo T (2021). Esophageal large cell neuroendocrine carcinoma. Dig Liver Dis.

[7] Galanis I, Simou M, Floros G (2022). Large-cell esophageal neuroendocrine carcinoma: report of a rare case. Cureus.

[8] Nakao Y, Okino T, Yamashita YI, Taki K, Nakagawa S, Matsumoto K (2019). Case report of aggressive treatments for large-cell neuroendocrine carcinoma of the esophagus. Int J Surg Case Rep.

[9] Wilson CI, Summerall J, Willis I, Lubin J, Inchausti BC (2000). Esophageal collision tumor (Large cell neuroendocrine carcinoma and papillary carcinoma) arising in a Barrett esophagus. Arch Pathol Lab Med.

[10] Umar Z, Ilyas U, Otusile I, Landry I (2022). Large-cell esophageal neuroendocrine tumor leading to hepatorenal syndrome. Cureus.

[11] Ichimata S, Aoyagi D, Takehana T, Uehara T, Shiozawa S (2019). A case of large cell neuroendocrine carcinoma exhibiting rhabdoid features in the esophagogastric junction. Pathol Int.

[12] Kuriry H, Swied AM (2015). Large-cell neuroendocrine carcinoma of the esophagus: a case from Saudi Arabia. Case Rep Gastroenterol.

[13] Terada T (2011). Neuroendocrine carcinoma of the esophagus: a case report with immunohistochemical and molecular genetic analyses of KIT and PDGFRA. Med Oncol.

[14] Tustumi F, Takeda FR, Uema RH, Pereira GL, Sallum RA, Cecconello I (2017). Primary neuroendocrine neoplasm of the esophagus—report of 14 cases from a single institute and review of the literature. Arq Gastroenterol.

[15] Tomiyama T, Orino M, Nakamaru K, Tanaka T, Suzuki R, Okazaki T (2018). Esophageal large-cell neuroendocrine carcinoma with inconsistent response to treatment in the primary and metastatic lesions. Case Rep Gastroenterol.

[16] Vayzband V, Doukas S, Esparragoza P (2022). Esophageal neuroendocrine carcinoma: a case report and literature review. Cureus.

[17] Fukuchi M (2014). A case of large cell neuroendocrine carcinoma of the esophagogastric junction. Esophagus.

[18] Giannetta E, Guarnotta V, Rota F, de Cicco F, Grillo F, Colao A (2019). A rare rarity: neuroendocrine tumor of the esophagus. Crit Rev Oncol Hematol.

[19] National Cancer Institute. SEER Incidence Data, 1975–2019: National Cancer Institute: Surveillance, Epidemiology, and End Results Program; https://seer.cancer.gov/data/.

[20] Lee CG, Lim YJ, Park SJ, Jang BI, Choi SR, Kim JK (2014). The clinical features and treatment modality of esophageal neuroendocrine tumors: a multicenter study in Korea. BMC Cancer.

